# The association between VEGF genetic variations and the risk of bronchopulmonary dysplasia in premature infants: a meta-analysis and systematic review

**DOI:** 10.3389/fped.2024.1476180

**Published:** 2024-11-14

**Authors:** Mohammad Golshan-Tafti, Reza Bahrami, Seyed Alireza Dastgheib, Mohamad Hosein Lookzadeh, Seyed Reza Mirjalili, Maryam Yeganegi, Maryam Aghasipour, Amirmasoud Shiri, Ali Masoudi, Amirhossein Shahbazi, Sepideh Azizi, Mahmood Noorishadkam, Hossein Neamatzadeh

**Affiliations:** ^1^ Department of Pediatrics, Islamic Azad University of Yazd, Yazd, Iran; ^2^ Neonatal Research Center, Shiraz University of Medical Sciences, Shiraz, Iran; ^3^ Department of Medical Genetics, School of Medicine, Shiraz University of Medical Sciences, Shiraz, Iran; ^4^ Mother and Newborn Health Research Center, Shahid Sadoughi University of Medical Sciences, Yazd, Iran; ^5^ Department of Obstetrics and Gynecology, Iranshahr University of Medical Sciences, Iranshahr, Iran; ^6^ Department of Cancer Biology, College of Medicine, University of Cincinnati, Cincinnati, OH, United States; ^7^ General Practitioner, School of Medicine, Shiraz University of Medical Sciences, Shiraz, Iran; ^8^ General Practitioner, Shahid Sadoughi University of Medical Sciences, Yazd, Iran; ^9^ Student Research Committee, School of Medicine, Ilam University of Medical Sciences, Ilam, Iran; ^10^ Shahid Akbarabadi Clinical Research Development Unit, Iran University of Medical Sciences, Tehran, Iran

**Keywords:** bronchopulmonary dysplasia, VEGF, polymorphism, premature infants, meta-analysis, genetic variations

## Abstract

**Objective:**

Previous studies on the link between VEGF gene polymorphisms and bronchopulmonary dysplasia (BPD) have yielded inconsistent results. This meta-analysis sought to clarify the relationship between genetic variations in the VEGF gene and the risk of BPD.

**Methods:**

Data were collected from multiple databases, including PubMed, Scopus, EMBASE, and CNKI, up to January 5, 2024.

**Results:**

Nineteen case-control studies were analyzed, featuring 1,051 BPD cases and 1,726 healthy neonates. The analysis included four studies on the −460T/C polymorphism (312 cases, 536 controls), four on the −2578C/A polymorphism (155 cases, 279 controls), six on the +405G/C polymorphism (329 cases, 385 controls), and five on the +936C/T polymorphism (225 cases, 526 controls). The meta-analysis suggests that the −460T/C polymorphism may protect against BPD (C vs. T: OR = 0.715, 95% CI 0.543–0.941, *p* = 0.017; CC vs. TT: OR = 0.478, 95% CI 0.233–0.983, *p* = 0.045; CC vs. CT + TT: OR = 0.435, 95% CI 0.248–0.764, *p* = 0.004). No significant associations were found between the −2578C/A, +405G/C, and +936C/T polymorphisms and BPD susceptibility.

**Conclusions:**

This meta-analysis indicates that the C allele of the −460T/C polymorphism may offer protection against BPD. No significant associations were observed for the −2578C/A, +405G/C, and +936C/T polymorphisms.

## Introduction

Bronchopulmonary dysplasia (BPD) is a common respiratory disorder found in premature infants, marked by specific clinical signs, imaging and histological features, and significant long-term effects (1, 2). This condition primarily affects premature infants, particularly those requiring mechanical ventilation and oxygen therapy, leading to extended hospitalization, frequent readmissions, and the necessity for specialized medical care (3, 4). BPD compromises respiratory function, causing difficulties in breathing, increased vulnerability to respiratory infections, and a reduced quality of life (5, 6). Since the original characterization of classic BPD by Northway et al. in 1967, the definition and diagnostic criteria have evolved, yet the diagnosis still fundamentally hinges on clinical signs related to oxygen dependence (7, 8). The clinical manifestations and prognosis of BPD, which arise from various etiologies, display significant variability, alongside notable differences in their underlying pathophysiological and molecular mechanisms (1, 9, 10). In recent years, improvements in survival rates among premature infants with restricted gestational age have led to a rising incidence of BPD, considerably affecting the quality of life for these children (11, 12). Epidemiological studies indicate that birth weight and gestational age are the most reliable predictors for BPD (13). Reports show that the prevalence of BPD in premature infants born before 30 weeks gestation ranges from 20% to 30% (14), while those weighing less than 1,500 g have a prevalence of 25%–35% (15). Similarly, infants born between 22 and 28 weeks gestation show an incidence of 20%–30% (1). Nearly 40% of preterm infants are at risk for developing BPD, with surviving children susceptible to long-term sequelae such as delayed language development, cerebral palsy, and cognitive impairments (16–18). Current treatment strategies for BPD mainly focus on respiratory support, nutritional optimization, and managing complications like infections and pulmonary hypertension (19, 20). Additionally, emerging therapies, including stem cell and gene therapy, are being explored as potential treatment options for BPD (21).

The pathogenesis of BPD is influenced by a variety of factors, including fetal infection or inflammation, prenatal steroid deficiency, oxidative stress, postnatal inflammation or infection, malnutrition, abnormal growth factor signaling, and genetic predisposition (22–24). Bhandari et al. first proposed the potential role of genetic factors in BPD development (25), with studies indicating that genetic factors significantly contribute to BPD susceptibility, as shown in twin studies where they accounted for 53%–82% of the risk (26). Understanding the underlying mechanisms is essential for tailoring treatments and predicting outcomes in BPD (10). The condition involves various biological processes, including local and systemic inflammatory responses, tissue repair, metabolism, and alterations in bacterial flora (27–31). Impaired pulmonary vascular growth, a key process in lung development, characterizes BPD, which has seen an increasing incidence over the years (32–34). Vascular endothelial growth factor (VEGF) plays a central role in angiogenesis and vascular permeability, significantly affecting lung development and function, particularly in the later stages of fetal lung maturation. Disruptions in the VEGF signaling pathway can result in abnormal blood vessel formation and lung development, heightening the risk of BPD (35–38). Alveolar type II epithelial cells are the primary producers of VEGF, which is vital for endothelial cell migration, survival, proliferation, and differentiation. VEGF is the critical regulatory factor for pulmonary blood vessel growth and maintenance during embryonic, fetal, and postnatal periods (39–42). Additionally, VEGF is implicated in hyperoxic lung injury in neonates (41, 43–45). Research shows that VEGF expression levels in premature infants and animal models of BPD induced by high oxygen can be significantly reduced compared to control groups at various time points (41, 46, 47). Evidence suggests that virus-mediated gene therapy or intramuscular injection of exogenous VEGF can promote pulmonary vessel development and enhance the radial alveolar count (RAC) in experimental BPD models; however, excessive VEGF expression may lead to pulmonary edema, hemorrhage, and other adverse effects (41, 48, 49).

The VEGF gene, situated on chromosome 6p21 and comprising 8 exons and 7 introns, has garnered significant research attention concerning its correlation with BPD in diverse populations (50). While the precise mechanisms by which these polymorphisms influence BPD onset remain unclear, it is posited that variations in the VEGF gene may impact its expression, potentially disrupting lung vascular development and function. Key characteristics of BPD include inadequate lung vascularization and abnormal alveolarization, and alterations in VEGF expression could impede these vital processes, leading to the condition's emergence (51, 52). Research suggests that reduced or absent VEGF expression, along with specific genetic variations, correlates with the incidence of BPD (41). A study from 2008 identified the −460T>C polymorphism in the VEGF gene as a potential risk factor for developing BPD (51). Moreover, research on a Chinese population indicated a significant association between the −2578C/A polymorphism and increased susceptibility to BPD (53). Additionally, findings from a Turkish cohort of premature infants demonstrated correlations between specific VEGF gene mutations and BPD onset (54). These findings imply that genetic variations within the VEGF gene may play a crucial role in predisposing individuals to BPD. Contrastingly, some studies have yielded inconclusive results, with one failing to establish a link between genetic polymorphisms at this site and BPD incidence in the Chinese population [48]. Similarly, Kwinta et al. reported no significant association between VEGF polymorphisms and BPD (51), and Filonzi et al. found no substantial correlation in their study of Italian VLBW newborns (50). Given the recognition of multiple polymorphic sites within the VEGF gene and observed ethnic and regional variations in gene distribution, this meta-analysis aimed to clarify the relationship between VEGF polymorphisms and the development of BPD in premature infants.

## Materials and methods

### Search strategy

The research methodology involved a comprehensive and systematic exploration of various online databases to collect pertinent literature, including prominent platforms such as PubMed, Web of Science, Europe PMC, ResearchGate, Elsevier, Cochrane Library, EMBASE, and SciELO. Additionally, databases specific to Chinese medical literature were utilized, including Wanfang Data, Chaoxing, the Chinese Medical Citation Index (CMCI), VIP Information Consulting, Chinese Medical Current Contents (CMCC), the Chinese Biomedical Database (CBD), Sinomed, MEDREX, and the China/Asia On Demand (CAOD)/Asia Document Delivery service. The Weipu Periodical Database from the Chinese National Knowledge Infrastructure (CNKI) was also included. The search, conducted with a cutoff date of January 5, 2024, specifically targeted case-control studies examining the association between VEGF polymorphisms and BPD. A carefully crafted search string incorporated keywords related to BPD, such as “Bronchopulmonary Dysplasia,” “Infant,” “Neonates,” “Preterm,” “Chronic Lung Disease’’ and terms associated with VEGF, including “Vascular endothelial growth factor,” “VEGF,” “Gene,” “Genetic,” “Single-Nucleotide Polymorphism,” and “Allele.” To enhance comprehensiveness, a manual review of references from relevant articles and reviews was conducted to identify additional studies not captured in the database search, ensuring an exhaustive literature review encompassing both English and Chinese publications. Importantly, informed consent was not required for this meta-analysis, as the study focused solely on analyzing existing literature, thus adhering to ethical guidelines regarding participant consent. This methodological rigor aimed to establish a solid foundation for understanding the potential relationship between VEGF polymorphisms and BPD in preterm infants and related populations.

### Inclusion and exclusion criteria

All studies included in this meta-analysis were required to adhere to specific criteria. They needed to be designed as either case-control or cohort studies, published in English or Chinese, and focus on the association between VEGF genetic variations and the susceptibility to BPD. The case group was defined as premature infants diagnosed with BPD, while the control group comprised premature infants without BPD. Additionally, studies had to provide sufficient and accessible data to calculate odds ratios (ORs) and 95% confidence intervals (CIs). Exclusion criteria encompassed case reports, case series, editorial correspondence, reviews, animal studies, *in vitro* experiments, conference abstracts, and other meta-analyses. Studies with incomplete data or inaccessible original manuscripts were also excluded, as were those with insufficient data for meaningful analysis. Furthermore, research involving family members, including family studies, sibling studies, and investigations of monozygotic or dizygotic twins, was not considered. Ultimately, any duplicate data or repetitious studies were disregarded.

### Data extraction

Two researchers conducted a systematic review by evaluating relevant study bibliographies according to strict inclusion and exclusion criteria. They collected and cross-validated data for accuracy and reliability, resolving disagreements through discussions or by consulting a third scientist. The review began with assessing titles and abstracts to exclude irrelevant studies, followed by a thorough examination of full texts for eligibility confirmation. Key data extracted included the primary author's name, publication year, country of research, participant ethnicity, genotyping techniques, total cases and controls, and genotype frequencies for individuals diagnosed with BPD and healthy controls. Important details such as Hardy-Weinberg equilibrium (HWE) test results and minor allele frequencies (MAFs) in non-BPD infants were also included to enhance the understanding of VEGF polymorphisms. To prevent redundancy from multiple studies by the same authors, only the most recent or largest sample size publication was considered. This structured approach demonstrates a strong commitment to producing a comprehensive literature review, deepening the understanding of the links between VEGF polymorphisms and BPD.

### Statistical analysis

The HWE was evaluated using the Chi-Square (*χ*²) statistic among healthy subjects in each study, with a significance threshold of *p* < 0.05. This assessment is vital as it verifies that the genetic variations in the study population align with expected frequencies under equilibrium, a necessary condition for dependable genetic association analyses. To investigate the relationship between VEGF polymorphisms and BPD risk, ORs and accompanying 95% CIs were calculated. The ORs serves as an indicator of the association between specific genetic variants and the likelihood of developing BPD, highlighting potential genetic risk factors. Statistical significance of the overall data was determined through a *Z*-test, which contrasts the population mean with the sample mean, a method apt for meta-analysis where results from multiple studies yield more robust conclusions. The meta-analysis considered five distinct genetic models: the allelic model (B vs. A), homozygote model (BB vs. AA), heterozygote model (BA vs. AA), dominant model (BB + BA vs. AA), and recessive model (BB vs. BA + AA). The Chi-square test, a common method for assessing study heterogeneity, was utilized, with significant heterogeneity indicated by *p* < 0.05. The I² statistic quantified heterogeneity, with values exceeding 50% prompting the use of a random-effects model (DerSimonian-Laird method) for conservative effect estimates, while a fixed-effect model (Mantel-Haenszel method) was applied when heterogeneity was low (*I*² ≤ 50%). Understanding the implications of fixed vs. random effects models is critical for accurate data analysis, with fixed effects appropriate for constant, time-invariant heterogeneity, and random effects accommodating random variations across studies. Model selection considerations highlight the necessity of accounting for unobserved heterogeneity, as this can bias random effects estimates if correlated with predictors. The chosen model's validity can be reinforced through tests like the Hausman test, guiding researchers in selecting suitable methodologies. Sensitivity analysis was conducted by systematically excluding one study at a time to evaluate the robustness of the findings, identifying studies that may unduly influence effect size estimates, particularly when substantial changes occur upon exclusion. Reporting shifts in effect sizes or directions is crucial for transparency and helps inform the reliability of conclusions regarding VEGF polymorphisms and BPD risk. Publication bias was assessed using Begg's and Egger's tests, along with a visual inspection of funnel plots for asymmetry; if bias was detected, the trim-and-fill method was applied for adjustments. Data synthesis of the primary studies was performed using the Comprehensive Meta-Analysis (Version 4.0) software (Biostat, USA), with statistical significance defined as a two-tailed *p*-value of less than 0.05.

## Results

### Characteristics of eligible studies

**Figure 1:**
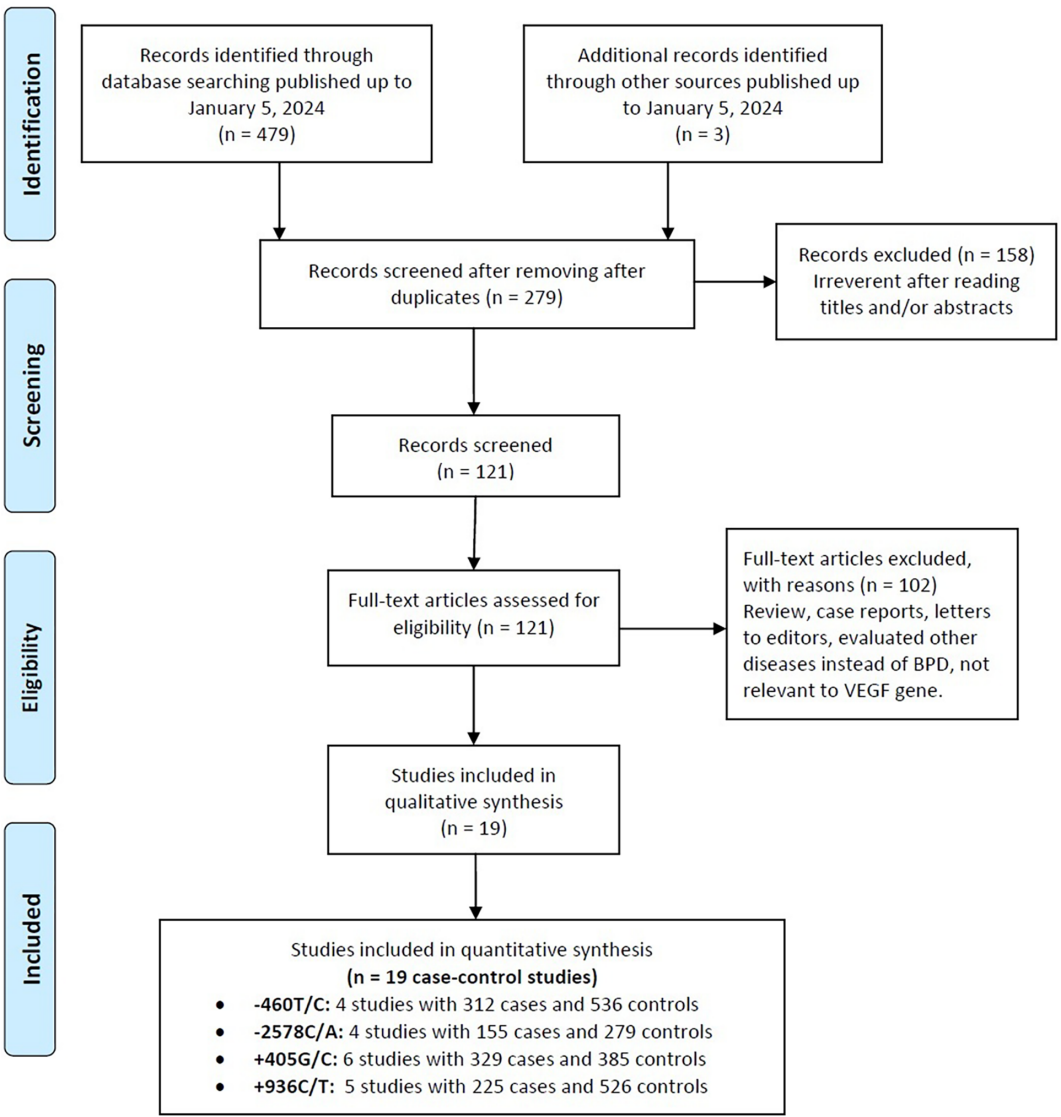
Flowchart depicting the selection process for studies included in the current meta-analysis.

Figure 1 presents a flowchart detailing the methodology for selecting studies for this analysis. An initial search across various online databases yielded 482 articles. After removing duplicates, 203 unique articles were identified. A review of titles and abstracts excluded 158 studies that did not meet the inclusion criteria, resulting in 68 for full-text assessment. This thorough review eliminated another 102 studies due to issues such as inappropriate control groups, insufficient data, and a focus on different VEGF gene polymorphic sites. Ultimately, 19 case-control studies from eight publications (50, 51, 53, 55–59) were included, representing 1,051 cases of BPD and 1,726 healthy neonates. Table 1 encapsulates the specifics of these studies, emphasizing certain VEGF gene polymorphisms: the −460T/C polymorphism was examined in four studies (312 cases, 536 controls), −2578C/A in four studies (155 cases, 279 controls), +405G/C in six studies (329 cases, 385 controls), and +936C/T in five studies (225 cases, 526 controls).

**Table 1:** Characteristics of the studies included in the meta-analysis.

First author/year	Ethnicity (Country)	Genotyping methods	Case/control	Patients					Healthy control					MAFs	HWE
				Genotypes			Alleles		Genotypes			Alleles			
−460T/C				TT	TC	CC	T	C	TT	TC	CC	T	C		
Bányász (2006) (55)	Hungary (Caucasian)	PCR-RFLP	23/102	6	13	4	25	21	23	53	26	99	105	0.515	0.685
Kwinta (2008) (60)	Poland (Caucasian)	PCR-RFLP	118/63	28	59	31	115	121	12	21	30	45	81	0.643	0.029
Poggi (2014) (56)	Italy (Caucasian)	TaqMan	71/271	24	47				76	195				NA	NA
Qiaoyan (2018) (57)	China (Asian)	PCR	100/100	38	62	0	138	62	29	71	0	129	71	0.355	≤0.001
−2578C/A				CC	CA	AA	C	A	CC	CA	AA	C	A		
Bányász (2006) (55)	Hungary (Caucasian)	PCR-RFLP	23/102	3	15	5	21	25	28	52	22	108	96	0.471	0.815
Mailaparambil (2010) (58)	Germany (Caucasian)	PCR-RFLP	46/107	9	19	18	37	55	31	57	19	119	95	0.444	0.413
Yan (2019) (53)	China (Asian)	PCR	36/14	29	5	2	63	9	6	5	3	17	11	0.393	0.347
Qiaoyan (2021) (57)	China (Asian)	PCR	50/56	8	12	30	28	72	18	18	20	54	58	0.518	0.007
+405G/C				GG	GC	CC	G	C	GG	GC	CC	G	C		
Bányász (2006) (55)	Hungary (Caucasian)	PCR-RFLP	23/102	13	9	1	35	11	47	48	7	142	62	0.304	0.257
Kwinta (2008) (60)	Poland (Caucasian)	PCR-RFLP	118/63	63	40	15	166	70	35	19	9	89	37	0.294	0.030
Mailaparambil (2010) (58)	Germany (Caucasian)	PCR-RFLP	47/108	27	15	5	69	25	50	43	15	143	73	0.338	0.251
Fujioka (2014) (59)	Japan (Asian)	Sequencing	55/52	4	29	22	37	73	10	22	10	42	42	0.500	0.757
Yan (2019) (53)	China (Asian)	PCR	36/14	19	12	5	50	22	7	3	4	17	11	0.393	0.039
Qiaoyan (2021) (57)	China (Asian)	PCR	50/56	5	30	15	40	60	12	28	16	52	60	0.536	0.969
+936C/T				CC	CT	TT	C	T	CC	CT	TT	C	T		
Mailaparambil (2010) (58)	Germany (Caucasian)	PCR-RFLP	46/108	37	8	1	82	10	85	23	0	193	23	0.106	0.215
Fujioka (2014) (59)	Japan (Asian)	Sequencing	55/42	27	27	1	81	29	26	15	1	67	17	0.202	0.491
Poggi (2014) (56)	Italy (Caucasian)	TaqMan	71/271	56	15				184	87				NA	NA
Qiaoyan (2021) (57)	China (Asian)	PCR	50/56	17	11	22	45	55	21	15	20	57	55	0.491	0.005
Filonzi (2022) (50)	Italy (Caucasian)	PCR-RFLP	33/49	24	8	1	56	10	34	14	1	82	16	0.163	0.748

Boldface denotes heterozygous + mutant homozygous.

The studies, published between 2007 and 2022, were accessible in English and Chinese, contributing to their reach. They included 11 studies on Caucasian populations and eight on Asian populations, enhancing the findings’ generalizability. Analyzing genotype distribution—specifically TT, TC, and CC frequencies among cases and controls—can elucidate associations with susceptibility or resilience to health conditions. Research by Bányász (55) identified significant variability in these distributions, likely influenced by environmental factors or population-specific traits. Genetic differences between Caucasian and Asian populations may arise from evolutionary pressures, environmental interactions, and genetic drift. The sample sizes in these analyses are critical; for instance, Poggi (56) had 271 controls compared to 14 in Yan (53), significantly impacting correlation and statistical significance. The genotyping methods used—ranging from PCR and PCR-RFLP to direct sequencing and TaqMan assays—underscore methodological rigor. Notably, all but two studies adhered to HWE in their control groups, essential for accurate genetic analysis interpretation.

### Included studies quality

The meta-analysis revealed significant variability in study quality, influenced by factors such as sample size, HWE, and genotyping methods. Fujioka (2014) was identified as the highest quality study, employing sequencing methods and adhering closely to HWE. Studies such as Bányász (2006), Poggi (2014), Mailaparambil (2010), and Yan (2019) were rated as moderate quality due to acceptable HWE *p*-values and reliable genotyping methods, despite some limitations like smaller sample sizes or imbalances between cases and controls. In contrast, Kwinta (2008), Qiaoyan (2018), and Qiaoyan (2021) were deemed low quality due to significant HWE deviations and inadequate sample sizes. Overall, while some studies provide reliable findings, others raise concerns that could affect the validity of the meta-analysis results.

### Quantitative data synthesis

**Figure 2:**
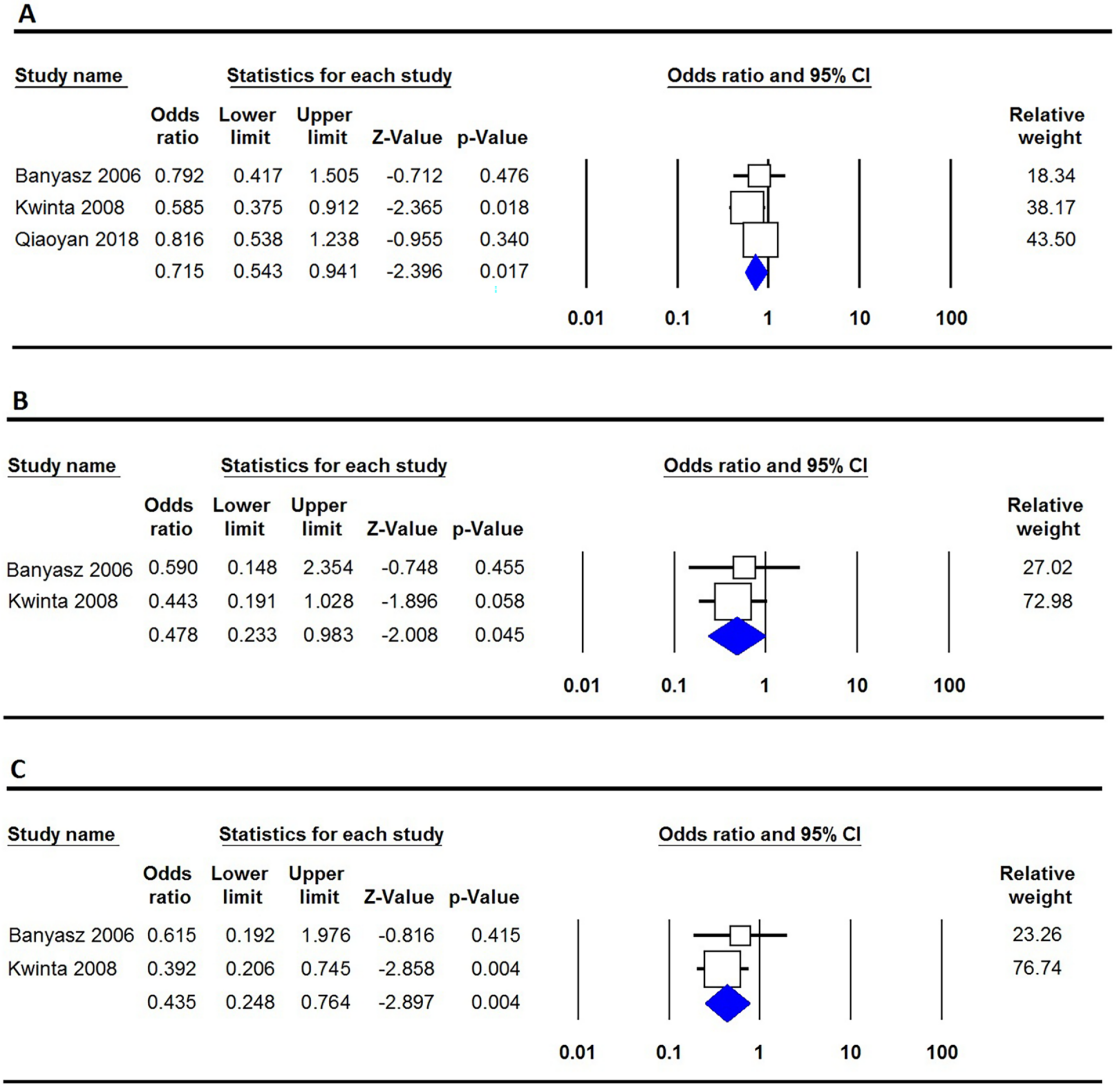
Forest plot illustrating the correlation between the VEGF −460T/C polymorphism and the risk of BPD. **(A)** Allele model (C vs. T); **(B)** Homozygote (CC vs. TT); **(C)** Recessive (CC vs. CT + TT).

Table 2 provides an analysis of the relationship between VEGF polymorphisms and the susceptibility to BPD. The results show that the −460T/C polymorphism offers a protective effect against BPD in three genetic models: the allele model (C vs. T: OR = 0.715, 95% CI 0.543–0.941, *p* = 0.017, Figure 2A), the homozygote model (CC vs. TT: OR = 0.478, 95% CI 0.233–0.983, *p* = 0.045, Figure 2B), and the recessive model (CC vs. CT + TT: OR = 0.435, 95% CI 0.248–0.764, *p* = 0.004, Figure 2C). In contrast, the −2578C/A, +405G/C, and +936C/T polymorphisms showed no significant association with BPD susceptibility in neonates.

**Table 2:** Meta-analysis results on the association between VEGF polymorphism and BPD risk.

	Genetic model	Type of model	Heterogeneity		Odds ratio				Publication Bias	
			*I*^2^ (%)	P_H_	OR	95% CI	Z_OR_	P_OR_	P_Beggs_	P_Eggers_
−460T/C	C vs. T	Fixed	0.00	0.529	0.715	0.543–0.941	−2.396	0.017	1.000	0.898
	CC vs. TT	Fixed	0.00	0.729	0.478	0.233–0.983	−2.008	0.045	NA	NA
	CT vs. TT	Fixed	0.00	0.513	0.831	0.534–1.292	−0.822	0.411	1.000	0.475
	CC + CT vs. TT	Fixed	0.00	0.981	0.735	0.524–1.031	−1.785	0.074	0.734	0.447
	CC vs. CT + TT	Fixed	0.00	0.507	0.435	0.248–0.764	−2.897	0.004	NA	NA
-2578C/A	A vs. C	Random	81.99	0.001	1.196	0.558–2.564	0.459	0.646	0.308	0.069
	AA vs. CC	Random	65.55	0.033	1.727	0.571–5.227	0.967	0.333	0.308	0.105
	AG vs. CC	Fixed	55.24	0.082	1.134	0.639–2.013	0.431	0.667	1.000	0.606
	AA + AC vs. CC	Random	73.48	0.010	1.253	0.441–3.563	0.423	0.672	0.734	0.454
	AA vs. AC + CC	Random	63.71	0.041	1.552	0.660–3.653	1.007	0.314	0.089	0.014
Caucasian	A vs. C	Fixed	0.00	0.426	1.646	1.111–2.438	2.487	0.013	NA	NA
	AA vs. CC	Fixed	0.00	0.643	2.879	1.258–6.591	2.503	0.012	NA	NA
	AG vs. CC	Fixed	7.963	0.297	1.507	0.714–3.182	1.077	0.282	NA	NA
	AA + AC vs. CC	Fixed	0.00	0.603	1.894	0.937–3.827	1.779	0.075	NA	NA
	AA vs. AC + CC	Fixed	59.88	0.114	2.081	1.107–3.914	2.275	0.023	NA	NA
Asian	A vs. C	Random	93.62	≤0.001	0.757	0.073–7.814	−0.234	0.815	NA	NA
	AA vs. CC	Random	87.30	0.005	0.770	0.034–17.508	−0.164	0.870	NA	NA
	AG vs. CC	Random	76.54	0.039	0.598	0.086–4.147	−0.520	0.603	NA	NA
	AA + AC vs. CC	Random	89.80	0.002	0.702	0.054–9.139	−0.270	0.787	NA	NA
	AA vs. AC + CC	Random	82.54	0.017	0.893	0.076–10.422	−0.091	0.928	NA	NA
+405G/C	C vs. G	Random	44.58	0.108	1.041	0.818–1.324	0.327	0.744	1.000	0.553
	CC vs. GG	Fixed	45.97	0.099	1.136	0.678–1.905	0.484	0.628	1.000	0.972
	CG vs. GG	Fixed	37.19	0.158	1.109	0.756–1.627	0.530	0.596	0.259	0.198
	CC + CG vs. GG	Fixed	48.52	0.084	1.041	0.732–1.481	0.224	0.823	0.259	0.261
	CC vs. CG + GG	Fixed	0.00	0.441	1.020	0.665–1.564	0.090	0.928	0.259	0.228
Caucasian	C vs. G	Fixed	0.00	0.562	0.838	0.608–1.154	−1.083	0.279	1.000	0.540
	CC vs. GG	Fixed	0.00	0.807	0.754	0.383–1.483	−0.818	0.414	1.000	0.472
	CG vs. GG	Fixed	0.00	0.457	0.840	0.538–1.311	−0.768	0.442	1.000	0.543
	CC + CG vs. GG	Fixed	0.00	0.459	0.815	0.541–1.229	−0.975	0.330	1.000	0.530
	CC vs. CG + GG	Fixed	0.00	0.944	0.794	0.414–1.528	−0.688	0.491	0.296	0.333
Asian	C vs. G	Fixed	47.64	0.148	1.379	0.958–1.987	1.728	0.084	1.000	0.434
	CC vs. GG	Fixed	63.23	0.066	2.018	0.906–4.493	1.718	0.086	1.000	0.534
	CG vs. GG	Fixed	0.00	0.729	2.449	1.155–5.193	2.335	0.020	1.000	0.467
	CC + CG vs. GG	Fixed	31.72	0.231	2.080	1.043–4.150	2.080	0.038	1.000	0.888
	CC vs. CG + GG	Fixed	46.02	0.157	1.229	0.698–2.163	0.715	0.475	1.000	0.459
+936C/T	T vs. C	Fixed	0.00	0.852	1.188	0.844–1.672	0.985	0.325	0.308	0.264
	TT vs. CC	Fixed	0.00	0.803	1.460	0.668–3.190	0.949	0.343	0.308	0.563
	TC vs. CC	Fixed	0.00	0.552	1.047	0.659–1.663	0.194	0.846	0.734	0.261
	TT + TC vs. CC	Fixed	16.45	0.310	0.911	0.640–1.296	−0.518	0.604	1.000	0.318
	TT vs. TC + CC	Fixed	0.00	0.765	1.476	0.726–2.999	1.076	0.282	0.308	0.676
Asian	T vs. C	Fixed	0.00	0.733	1.230	0.841–1.798	1.066	0.286	1.000	0.535
	TT vs. CC	Fixed	0.00	0.973	1.326	0.592–2.967	0.686	0.493	0.296	0.603
	TC vs. CC	Fixed	0.00	0.446	1.156	0.673–1.987	0.526	0.599	0.296	0.059
	TT + TC vs. CC	Fixed	0.00	0.566	1.230	0.751–2.013	0.823	0.411	1.000	0.479
	TT vs. TC + CC	Fixed	0.00	0.914	1.362	0.658–2.817	0.832	0.405	1.000	0.582

The analysis considered ethnic variations, particularly among Caucasians and Asians, to explore differences in the relationship between VEGF polymorphisms and BPD susceptibility. In Caucasian neonates, a significant association between the −2578C/A polymorphism and BPD risk was found across several genetic models. In the allele model, the A allele was linked to a higher BPD risk compared to the C allele (OR = 1.646, 95% CI 1.111–2.438, *p* = 0.013). The homozygote model showed that individuals with the AA genotype had an increased risk of BPD compared to those with the CC genotype (OR = 2.879, 95% CI 1.258–6.591, *p* = 0.012). The recessive model indicated that neonates with the AA genotype were at greater BPD risk than those with AC or CC genotypes (OR = 2.081, 95% CI 1.107–3.914, *p* = 0.023). In Asian neonates, a significant association was found for the +405G/C polymorphism under the heterozygote model, with CG genotype individuals at increased BPD risk compared to GG genotype individuals (OR = 2.449, 95% CI 1.155–5.193, *p* = 0.020). The dominant model further confirmed this, showing that individuals with the CC or CG genotype had a higher risk of BPD than those with the GG genotype (OR = 2.080, 95% CI 1.043–4.150, *p* = 0.038).

### Sensitivity analysis

The sensitivity analysis of VEGF genetic variations and BPD risk in premature infants revealed differing stability across genetic models. The −460T/C variant consistently yielded reliable results, aligning with the fixed-effect analyses. In contrast, the −2578C/A variant showed variability, suggesting instability and potential confounding factors. The +405G/C variant displayed stability in fixed models, reinforcing the findings, while the +936C/T variant produced consistent results across all comparisons, further validating the analysis. Excluding studies with controls deviating from HWE showed no significant heterogeneity before or after this exclusion, emphasizing the necessity of adhering to HWE in data interpretation. These findings highlight the complexities of genetic influences on BPD risk, with some variants demonstrating greater stability and confidence in their associations.

### Heterogeneity analysis

The analysis of VEGF genetic variations and their association with BPD risk in premature infants revealed varying levels of heterogeneity among polymorphisms. The −460T/C variant showed no heterogeneity, indicated by an *I*² of 0%. In contrast, the −2578C/A polymorphism displayed significant heterogeneity (*I*² = 81.99%) when comparing A vs. C in a random effects model; however, subgroup analyses found no heterogeneity in Caucasians (*I*² = 0%) and high heterogeneity in Asians (*I*² = 87.30% for AA vs. CC). The +405G/C variant had moderate heterogeneity overall (*I*² = 44.58%), with no heterogeneity in Caucasians (*I*² = 0%) and moderate heterogeneity in Asians (*I*² = 63.23% for CC vs. GG). Finally, the +936C/T polymorphism showed consistent results with an *I*² of 0%, indicating no heterogeneity. These findings highlight the variability in genetic influences on BPD risk, shaped by population differences and specific VEGF polymorphisms.

### Publication bias

**Figure 3:**
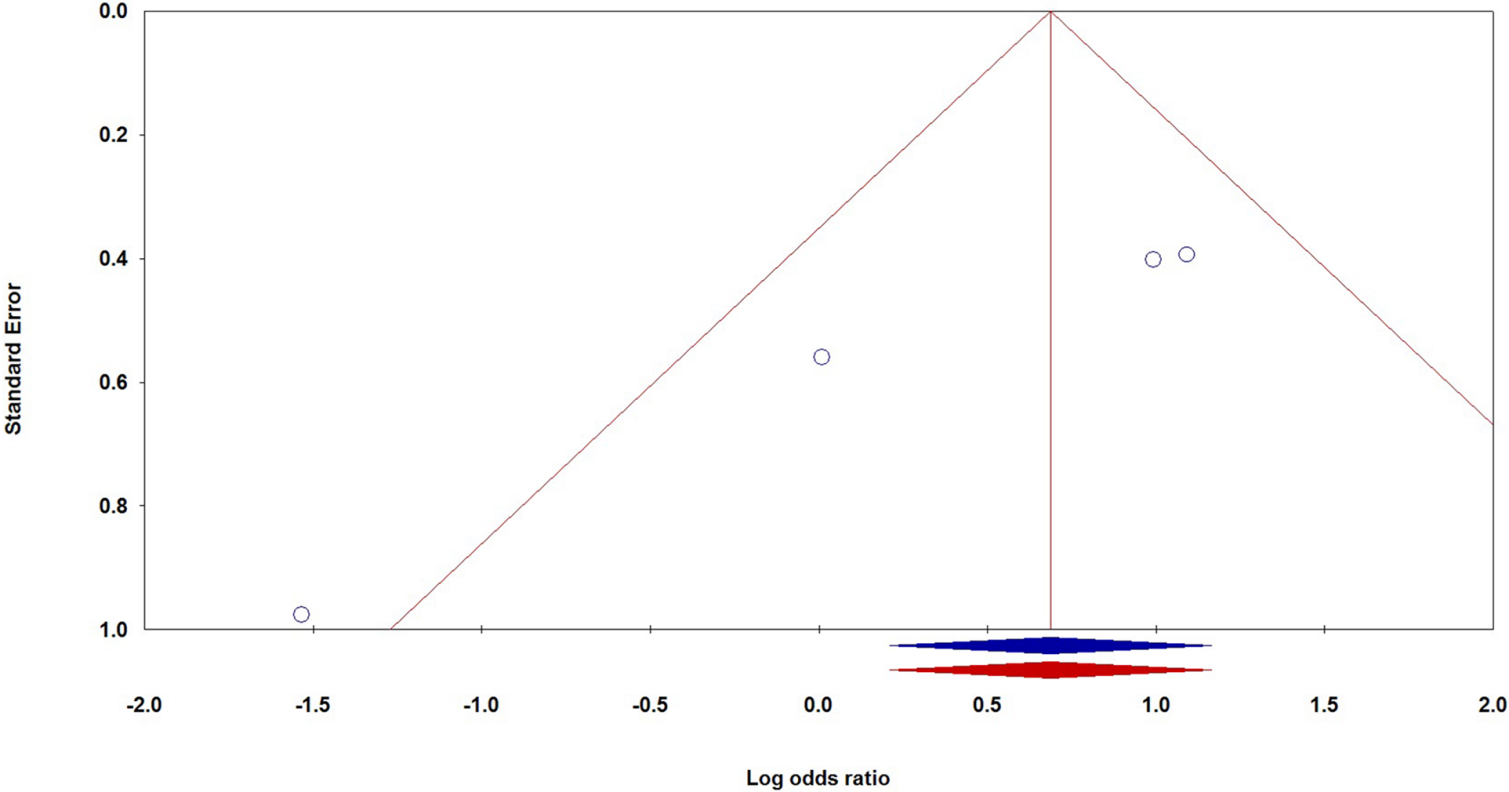
Begg's funnel plot for the publication bias test, examining the correlation between the VEGF −2578C/A polymorphism and the risk of BPD under the recessive model (AA vs. AC + CC).

The investigation into publication bias related to VEGF genetic variations and the risk of BPD in premature infants revealed varying outcomes depending on the specific genetic variants examined. For the −460T/C polymorphism, Egger's Test indicated potential publication bias, with a *p*-value of 0.045 in the CC vs. TT comparison. Additionally, significant bias was observed for the −2578C/A variant in the AA vs. AC + CC comparison (P_Eggers_ = 0.014), and similar biases were noted in the Caucasian subgroup for AA vs. CC (P_Eggers_ = 0.012) and in the Asian subgroup across multiple comparisons, including AA vs. AC + CC (P_Eggers_ = 0.014). Conversely, the +405G/C variant did not demonstrate significant bias, as most P_Eggers_ values were above 0.05. The +936C/T polymorphism showed consistent P_Eggers_ values exceeding 0.5, indicating a lack of significant publication bias. To address the publication bias associated with the −2578C/A polymorphism under the recessive model, we utilized the non-parametric “trim and fill” method developed by Duval and Tweedie. Our analysis showed that this method yielded results consistent with the original, reinforcing the statistical robustness of our findings. Figure 3 shows that Begg's funnel plot assessed publication bias by analyzing the correlation between the VEGF −2578C/A polymorphism and BPD risk using the recessive model (AA vs. AC + CC).

### HWE

HWE provides a foundational framework for evaluating genetic variation within a population. Deviations from HWE can indicate factors like selection pressures, inbreeding, migration, or population structure, all of which influence allele frequencies. This analysis identifies several instances of HWE deviations, particularly in the Polish population for the −460T/C variant, which has a significant *p*-value of 0.029. This indicates that allele frequencies in this population may be affected by factors beyond random mating, possibly reflecting selection pressures or non-random mating. In contrast, the Hungarian population for the same variant shows no significant deviation from HWE, with a *p*-value of 0.685, suggesting an equilibrium. Meanwhile, the Chinese population displays a notably low *p*-value (≤0.001) for the −460T/C variant, indicating a strong HWE deviation, potentially due to population structure or selection influences. Overall, the observed HWE deviations reveal significant variability across populations and variants, underscoring the importance of considering population-specific genetic factors in studying the relationship between genetic variants and health outcomes, such as BPD.

### MAF

Analyzing MAFs offers insights into genetic diversity and the role of specific variants in BPD susceptibility. This examination reveals significant MAF variation among different populations and genetic variants. For instance, the −460T/C variant has a high MAF in Poland (0.643), suggesting a potential link to demographic-specific phenotypes. In contrast, its MAF in China (0.355) reflects a lower prevalence, possibly due to varying evolutionary pressures or health profiles. Variations in MAF for the −2578C/A and +405G/C variants highlight the impact of ethnic background on genetic diversity. Discrepancies in MAF within Chinese groups for −2578C/A [e.g., 0.393 in Yan (2019) vs. 0.518 in Qiaoyan (2021)] may indicate temporal changes or differences in sampling methods. Notably, the MAF for the +936C/T variant is low in the German population (0.106) compared to the Chinese population (0.491), suggesting a higher likelihood of certain traits or susceptibilities in the Asian cohort. These variations emphasize the need to consider the genetic structure of each group when assessing risks for BPD. Future research should focus on population genetics to better understand how these genetic disparities influence health outcomes across diverse ethnicities.

## Discussion

Research on the −460T/C (rs833061) polymorphism of the VEGF gene in relation to BPD is limited. The T allele is associated with reduced VEGF promoter activity. Our analysis of four studies with 312 cases and 536 controls indicates that this polymorphism may protect against BPD. A study in China assessed 28 gene loci, including the −460T/C locus, and found no significant correlation with BPD incidence in premature infants (53). This contrasts with findings from a study by Kwinta et al. in the United States and results reported by Fujioka et al. in Japan (59), which align with another Chinese study by Kun et al. (61). Kwinta et al., in a prospective examination of gene polymorphism in 181 premature infants (average gestational age of 28 weeks), reported a 9% increased risk of BPD associated with the T allele of −460T/C, noting reduced risk for VEGF −460CC homozygotes compared to infants with −460TT or −460TC genotypes (60). Furthermore, a Japanese study employing multivariate logistic regression did not find any association between the −460T/C polymorphism of the VEGF gene and BPD occurrence. To understand the discrepancies in findings, the study compared genotype frequency distributions of this locus in Mongolian and Han premature infants in Inner Mongolia with relevant results from the U.S. and Japan. The analysis revealed a statistically significant difference in genotype frequency distribution compared to the U.S., but no significant difference compared to Japan, suggesting that variations in gene distributions among different populations and regions may account for the inconsistencies in research results (59).

Our pooled analysis of four studies involving 155 cases and 279 controls found no general link between the −2578C/A polymorphism and an increased risk of BPD. However, subgroup analyses revealed a significant association in Caucasian neonates across various genetic models, with the allele model indicating that the A allele was linked to a higher BPD risk compared to the C allele, yielding an odds ratio of 1.646 (95% CI 1.111–2.438, *p* = 0.013). A German study focusing on 155 preterm infants identified the −2578C/A polymorphism among three key genetic sites (−2578C/A, +405G/C, and +936C/T) and suggested a potential link to BPD. Outcomes related to this polymorphism varied by region and ethnicity. In 2019, Yan et al. reported potential associations between the +405G>C and −2578C>A polymorphisms in the VEGF gene and BPD onset. Their research in a Chinese cohort indicated that infants with the A allele were more likely to develop BPD compared to those with the CC genotype (53). Additionally, a Turkish population study suggested that the A allele may increase BPD susceptibility BPD (54). In contrast, some studies, including one on a Caucasian population, found no significant associations between the −2578C/A polymorphism and BPD risk, with no notable differences in genotype distributions between cases and controls (55).

A common polymorphism in the VEGFA gene, identified as +405G/C (rs2010963), was located near the 5′-untranslated region. Studies had shown that haplotypes containing the prevalent polymorphisms at position −460C within the promoter region, as well as at position +405G, exhibited a significant 71% increase in basal promoter activity compared to the wild-type sequence (62). Research indicated that individuals with the +405CC genotype had markedly higher VEGF serum levels compared to those with other genotypes of the same polymorphism. Specifically, the presence of the +405G allele appeared to reduce VEGF expression via the internal ribosome entry site and hinder translation of the larger L-VEGF isoform (63, 64). Additional investigations highlighted a significant correlation between VEGF protein production in lipopolysaccharide-stimulated peripheral blood mononuclear cells and the +405 polymorphism genotype, with +405G genotype carriers showing increased VEGF production (62, 65). Our meta-analysis of six studies involving 329 cases and 385 controls found no overall association between the +405G/C polymorphism and BPD risk. However, in Asian neonates, those with the CG genotype had a significantly higher risk of BPD compared to those with the GG genotype (OR = 2.449, 95% CI 1.155–5.193, *p* = 0.020). Additionally, the dominant model indicated that individuals with either the CC or CG genotype faced an increased risk of BPD relative to those with the GG genotype (OR = 2.080, 95% CI 1.043–4.150, *p* = 0.038). Two distinct studies by Mailaparambil et al. (58) and Kwinta et al. (51) found no significant association between the +405G/C polymorphism and the development of BPD in German and Polish newborns, respectively. In Hungary, a study investigated the correlation between functional genetic variations in VEGF and the likelihood of preterm birth or perinatal morbidity, concluding that carriers of the −2578 AA genotype had a decreased risk of acute renal failure (ARF). These results suggested that the VEGF +405G/C polymorphism may have been linked to an elevated risk of preterm birth, while the VEGF −2578C/A variant could have contributed to perinatal complications like necrotizing enterocolitis (NEC) and ARF, but not to BPD development (55).

The +936 C/T (rs3025039) genetic variation was situated within the 3′ UTR of the VEGF gene. Analysis of transcription factor binding sites revealed that this polymorphism eliminated a binding site for AP-4, a transcription factor that enhanced viral and cellular gene expression. A comprehensive analysis, combining data from five studies with 225 cases and 526 controls, found no significant association between the +936 C/T polymorphism and BPD risk in the general population or among Asian neonates. In 2013, Fujioka et al. investigated six VEGF polymorphisms, including −1498T/C, −1154G/A, −634C/G, −7C/T, +936C/T, and 1612G/A, in a Japanese cohort, establishing a causal link between VEGF-634C/G and BPD. Their stepwise logistic regression demonstrated that the G alleles of VEGF-634C/G, prolonged ventilation, and male gender were significant independent risk factors for BPD. They also noted strong linkage disequilibrium between −634C/G and several SNPs in the promoter and 5′ UTR, with a lower frequency of the −634G/−7C and +405 haplotypes in BPD patients compared to controls. However, they found no correlation between VEGF +936 C/T and BPD in this population (59). More recently, Filonzi et al. examined VEGFR1 −71° C/T and VEGF +936 C/T in a cohort of 33 BPD infants and 49 controls, finding no direct association at either the genotypic or allelic levels (50). In 2010, Mailaparambil et al. genotyped 155 infants to explore 37 gene variants and noted a potential link between certain VEGF polymorphisms and BPD, but no significant association was found for the +936 C/T variant (58).

Research on various polymorphisms of the VEGF gene and their association with BPD revealed significant inconsistencies and varying outcomes across different populations. For instance, the −460T/C polymorphism exhibited polarized results. Some studies suggested that the T allele may have conferred a protective effect against BPD, while others indicated an increased risk, particularly highlighted in the analysis by Kwinta et al., which found a 9% increased risk in premature infants carrying the T allele (51). Similarly, the −2578C/A polymorphism presented a nuanced relationship; a pooled analysis indicated no overall link to BPD, yet Caucasian neonates with the A allele appeared to be at a higher risk (58). Geographic and ethnic variations, such as those observed in Chinese and Turkish studies, further complicated the understanding of this relationship, as these populations reported differing susceptibilities. This underscored the importance of population-specific genetic epidemiology (53, 54). The +405G/C polymorphism suggested that elevated VEGF expression was associated with the CC genotype; however, findings were not uniformly replicated across studies in European populations, where no significant relationship with BPD was established (51, 55). Additionally, research concerning the +936C/T variant indicated that it may not have influenced BPD risk, as shown in analyses across diverse cohorts, including those within the Japanese population (59). These variances in genotype frequencies and outcomes highlighted the challenge of drawing definitive conclusions about VEGF genetic variability and BPD. This emphasized the need for further, more standardized studies across different populations to clarify these complex interactions and their clinical implications.

### Clinical significance: genetic insights into BPD susceptibility and tailored neonatal care

The findings on VEGF polymorphisms and their link to BPD susceptibility emphasize the crucial role of genetic factors in neonatal health, particularly for vulnerable groups like premature infants. The protective effect of the −460T/C polymorphism suggests potential early intervention strategies to prevent BPD. Clinically, recognizing specific VEGF polymorphisms could enhance risk assessments for high-risk infants, allowing genetic screening to identify those who may benefit from personalized therapies. For example, Caucasian infants with the AA genotype associated with the −2578C/A polymorphism may require closer monitoring and proactive management to prevent BPD. Understanding these genetic associations can guide pharmacogenomic strategies to optimize treatment based on the presence of protective alleles, possibly by exploring therapies that simulate the effects of the A allele of −460T/C. Variations in risk by ethnicity highlight the need for personalized medicine approaches, as genetic profiles influence BPD incidence and presentations. Clinicians must consider ethnic background in assessing risks and formulating management strategies. Ongoing research into how these polymorphisms affect VEGF and related pathways is critical for developing effective interventions. Insights from VEGF polymorphisms related to BPD can significantly enhance neonatal care by enabling personalized risk assessments and customized therapies, factoring in ethnic considerations. Screening for specific VEGF variants like −460T/C and −2578C/A allows healthcare professionals to identify infants at different risk levels for BPD, facilitating targeted monitoring and interventions. A thorough understanding of genetic predispositions to BPD can inform therapeutic strategies; infants with protective polymorphisms may need less respiratory support or may be suited for new therapies. The risk differences among ethnic groups indicate treatment protocols could be refined based on genetics, potentially influencing interventions like corticosteroid use. Improved genetic counseling can inform families about pregnancy risks associated with these polymorphisms. Identifying genetic markers may enhance predictions of treatment responses, allowing for tailored adjustments to optimize patient outcomes. Ultimately, these insights can propel research towards innovative therapeutic targets, improving prevention strategies and treatment choices for high-risk neonates. Integrating genetic insights into clinical practice can lead to better outcomes for at-risk neonates through personalized strategies and enhanced risk management aligned with individual genetic profiles. The connection between VEGF polymorphisms and BPD susceptibility offers a significant opportunity to incorporate genetic testing into neonatal care, aiming to reduce the incidence and severity of BPD in newborns.

### Limitaions

To the best of our knowledge, the present meta-analysis represents the initial aggregation of data regarding the role of VEGF polymorphisms in the development of BPD. This analysis was conducted by comprehensively searching the entire database of the network. However, there are several limitations in this study that should be acknowledged. Firstly, the sample sizes utilized in the present investigation were limited in size. Consequently, there exists the possibility of a type II error in relation to the restricted number of studies. Secondly, we exclusively incorporated published studies in the English language. Consequently, there exists the potential for relevant studies in alternative languages or unpublished studies, which could introduce selection bias. Thirdly, the genotypic distribution of controls in certain studies did not adhere to the HWE. Nonetheless, a sensitivity analysis demonstrated the stability of the estimations. Fourthly, the association between VEGF gene polymorphisms and the development of BPD suggests a genetic component to susceptibility. However, this genetic influence is often overshadowed by environmental and clinical factors, with gestational age and birth weight serving as significant determinants that increase BPD risk in premature infants, often more so than genetic predispositions. Other contributors include exposure to high oxygen levels and mechanical ventilation, which independently heighten lung injury risk, along with infections and inflammatory responses that complicate lung development. Maternal health issues such as diabetes, hypertension, and the quality of prenatal care and nutrition are critical in influencing BPD risk. Additionally, ethnic and socioeconomic factors can affect healthcare access and outcomes for preterm infants, further complicating the genetic relationship with BPD. While VEGF polymorphisms provide important insights into genetic susceptibility, their impact is likely limited compared to more direct and modifiable clinical factors, underscoring the necessity of addressing these high-risk elements for effective BPD prevention and management. It is also possible that specific confounding variables like gender, birth weight, and gestational age can modify the associations between VEGF gene polymorphisms and BPD risk; however, our inability to assess adjusted estimations for these confounders is due to a lack of data or inconsistent reporting. Finally, due to the intricate nature of the etiology of BPD, There is a possibility that there exists a contextual interaction between polymorphisms of the VEGF gene and other genetic variants, in addition to environmental factors, which were not investigated in the present study due to its limited scope.

## Conclusion

The meta-analysis indicates that the −460T/C genetic variant may provide a protective effect against the occurrence of BPD in preterm infants. Conversely, no significant associations were observed between the −2578C/A, +405G/C, and +936C/T genetic variations and the susceptibility to BPD in newborns. Furthermore, the study indicates complex interactions between VEGF genetic variants and BPD risk, revealing ethnic disparities. The protective influence of the −460T/C variant is counterbalanced by risks from specific polymorphisms found in Caucasian and Asian neonates, with the −2578C/A variant significantly correlating with increased BPD risk in Caucasians, and the +405G/C variant presenting substantial risks for Asian infants. These outcomes highlight the urgent need for additional research, especially within diverse populations, to enhance our understanding of the genetic factors affecting BPD risk and to inform the development of targeted prevention strategies. Addressing the limitations of this analysis, particularly the scarcity of data from a range of ethnic groups and small sample sizes, will be crucial for future investigations.

## Data Availability

The raw data supporting the conclusions of this article will be made available by the authors, without undue reservation.
